# *In vivo* monitoring of intracellular Ca^2+^ dynamics in the pancreatic β-cells of zebrafish embryos

**DOI:** 10.1080/19382014.2018.1540234

**Published:** 2018-12-06

**Authors:** Reka Lorincz, Christopher H. Emfinger, Andrea Walcher, Michael Giolai, Claudia Krautgasser, Maria S. Remedi, Colin G. Nichols, Dirk Meyer

**Affiliations:** a Institute of Molecular Biology/CMBI, University of Innsbruck, Innsbruck, Austria; b Department of Cell Biology and Physiology, Washington University School of Medicine, St. Louis, MO, USA; c Center for the Investigation of Membrane Excitability Diseases (CIMED), Washington University School of Medicine, St. Louis, MO, USA; d Department of Medicine, Washington University School of Medicine, St. Louis, MO, USA

**Keywords:** *cacna1c*, *Cav1.2* channel, early zebrafish development, GCaMP6s, glucose-sensing of beta cells, *in vivo* imaging

## Abstract

Assessing the response of pancreatic islet cells to glucose stimulation is important for understanding β-cell function. Zebrafish are a promising model for studies of metabolism in general, including stimulus-secretion coupling in the pancreas. We used transgenic zebrafish embryos expressing a genetically-encoded Ca^2+^ sensor in pancreatic β-cells to monitor a key step in glucose induced insulin secretion; the elevations of intracellular [Ca^2+^]_i_. *In vivo* and *ex vivo* analyses of [Ca^2+^]_i_ demonstrate that β-cell responsiveness to glucose is well established in late embryogenesis and that embryonic β-cells also respond to free fatty acid and amino acid challenges. *In vivo* imaging of whole embryos further shows that indirect glucose administration, for example by yolk injection, results in a slow and asynchronous induction of β-cell [Ca^2+^]_i_ responses, while intravenous glucose injections cause immediate and islet-wide synchronized [Ca^2+^]_i_ fluctuations. Finally, we demonstrate that embryos with disrupted mutation of the *Ca_V_1.2* channel gene *cacna1c* are hyperglycemic and that this phenotype is associated with glucose-independent [Ca^2+^]_i_ fluctuation in β-cells. The data reveal a novel central role of *cacna1c* in β-cell specific stimulus-secretion coupling in zebrafish and demonstrate that the novel approach we propose – to monitor the [Ca^2+^]_i_ dynamics in embryonic β-cells *in vivo* – will help to expand the understanding of β-cell physiological functions in healthy and diseased states.

## Introduction

Assessing the response of pancreatic islet cells to glucose stimulation is important for understanding β-cell function in healthy and diseased states. Until now, pancreatic β-cell physiology has been analyzed mainly in isolated cell and islet systems.^1–5^ Importantly, β-cells under these conditions likely exhibit different physiology when compared to cells in their natural *in vivo* environment. A key step in mammalian glucose-stimulated insulin secretion is the elevation of intracellular [Ca^2+^]_i_. Non-invasive *in vivo* imaging of [Ca^2+^]_i_ has recently been facilitated by transplantation of pancreatic islets into the anterior chamber of the eye or the kidney capsule of mice. Such real-time monitoring facilitates the study of islet physiology and vascularization longitudinally, and enables *in vivo* screening of novel drugs and treatments.^6^ Ultimately, however, it is desirable to incorporate *in vivo* imaging of native intracellular [Ca^2+^]_i_ without interfering with the complex paracrine signalling networks regulating islet activity in native tissue (for a review, see ref.^7^) Here we tested transgenic zebrafish embryos expressing a genetically encoded Ca^2+^ sensor in their β-cells as a potential model for corresponding non-invasive *in vivo* applications. Non-mammalian vertebrates such as zebrafish (*Danio rerio*) have become relevant alternative organisms to study diabetes and other metabolic diseases,^8^ due to ease of genetic manipulation, high reproduction rates, and ready access for *in vivo* imaging due to larval transparency. Importantly, pancreata in mammals and in zebrafish have conserved physiological endocrine and exocrine function, similar cellular architecture, and conserved expression and function of most developmental genes.^9^ Accordingly, the zebrafish has proven highly productive for studies of pancreas development,^10–12^ and regeneration.^13,14^ In mammals as well as in zebrafish the pancreas develops from the endodermal germ layer and later compromises endocrine and exocrine tissue.^15^ Within one day of development, the zebrafish embryo forms a single ‘primary’ pancreatic islet with ~60–70 mono-hormonal α-, β-, δ-, ϵ-cells. As development proceeds, the primary islet increases in size and additional secondary islets are formed, to adopt to growth-related requirements.

Glucose metabolism in zebrafish is also very similar to mammalian glucose metabolism, and overfed zebrafish displays obesity-related diabetes phenotypes including impaired glucose tolerance and increased insulin production.^16^ The molecular basis of glucose detection is well understood in mammalian pancreatic β-cells (for a review, see ref.^17^ and.^18^) Glucose is taken up by the facilitative glucose transporter (GLUT) GLUT2/SLC2A2, and is metabolized through glycolysis and oxidative phosphorylation, thereby generating adenosine triphosphate (ATP) and increasing the ATP/ADP ratio.^11,19^ The altered [ATP/ADP] ratio in the β-cell then leads to the closure of ATP-sensitive K^+^-channels (K_ATP_-channels), depolarization of the membrane, and consequent opening of voltage-dependent calcium channels (VDCCs).^20^ The influx of Ca^2+^ then triggers release of insulin from secretory granules.^21^ Orthologues of all major genes (GLUT, K_ATP_-channels and VDCCs) involved in mammalian glucose sensing and insulin secretion are also expressed in zebrafish, and show functional similarities.^22–25^ Studies suggest that glucose uptake in zebrafish, similar to mammals, occurs through GLUT transporters, with GLUT2 expression found in the endocrine pancreas of zebrafish larvae.^26,27^ Furthermore, we and others recently demonstrated that zebrafish islet β-cells express functional K_ATP_ channels with conserved structure and metabolic sensitivity to their mammalian counterparts, supporting the use of zebrafish as a model animal in islet glucose sensing and diabetes research.^28,29^

Excitability of β-cells has been investigated by multiple strategies including monitoring of the membrane potential by electrical recordings, and using Ca^2+^-sensitive dyes.^30–32^ More recently, genetically encoded Ca^2+^ indicators have been introduced as tools for non-invasive approaches to study excitable cells such as β-cells. The analysis of Ca^2+^ transients in corresponding models systems will help in understanding underlying causes of β-cell dysfunction (for example in the context of diabetes risk factors.^33,34^) *In vivo* imaging of [Ca^2+^]_i_ dynamics in transplanted (intraocular) mouse pancreatic islets showed reduction at a prediabetic stage, suggesting the potential of [Ca^2+^]_i_ as a functional marker to evaluate β-cells *in vivo* in diseased states.^35^ Nevertheless, the low litter numbers, complicated methodologies, high costs, and large experiment variability limit the use of mammalian species for such studies. Since genetic manipulation is easy and inexpensive in transparent zebrafish larvae, large-scale drug and genetic screens as well as fluorescent imaging applications for studying β-cell physiology in living animals are feasible.^36–38^ Monitoring of the glucose-stimulated Ca^2+^ influx in isolated juvenile and larvae zebrafish islets has been previously described.^39^ We recently generated a transgenic line expressing the genetically encoded Ca^2+^-sensor GCaMP6s, and in this study we have established protocols for *in vivo* monitoring of [Ca^2+^]_i_ dynamics in late embryonic animals. We found that β-cells in larvae show rare baseline spontaneous activities, but after intravenous injections of glucose, virtually all β-cells showed very rapid elevations in GCaMP6s fluorescence.

Additionally, we have used the GCaMP6s reporter to assess β-cell associated function of VDCCs. β-cell specific *Ca_V_1.2^−/-^* mice exhibit mild basal hyperglycemia, impaired glucose tolerance and lack of first-phase insulin secretion,^40,41^ but this important role in mammalian glucose-induced insulin secretion has not been assessed in zebrafish.^40–43^

## Results

### Zebrafish β-cell [Ca^2+^]_i_ responses depend on the route of glucose administration *in vivo*

**Figure 1.**
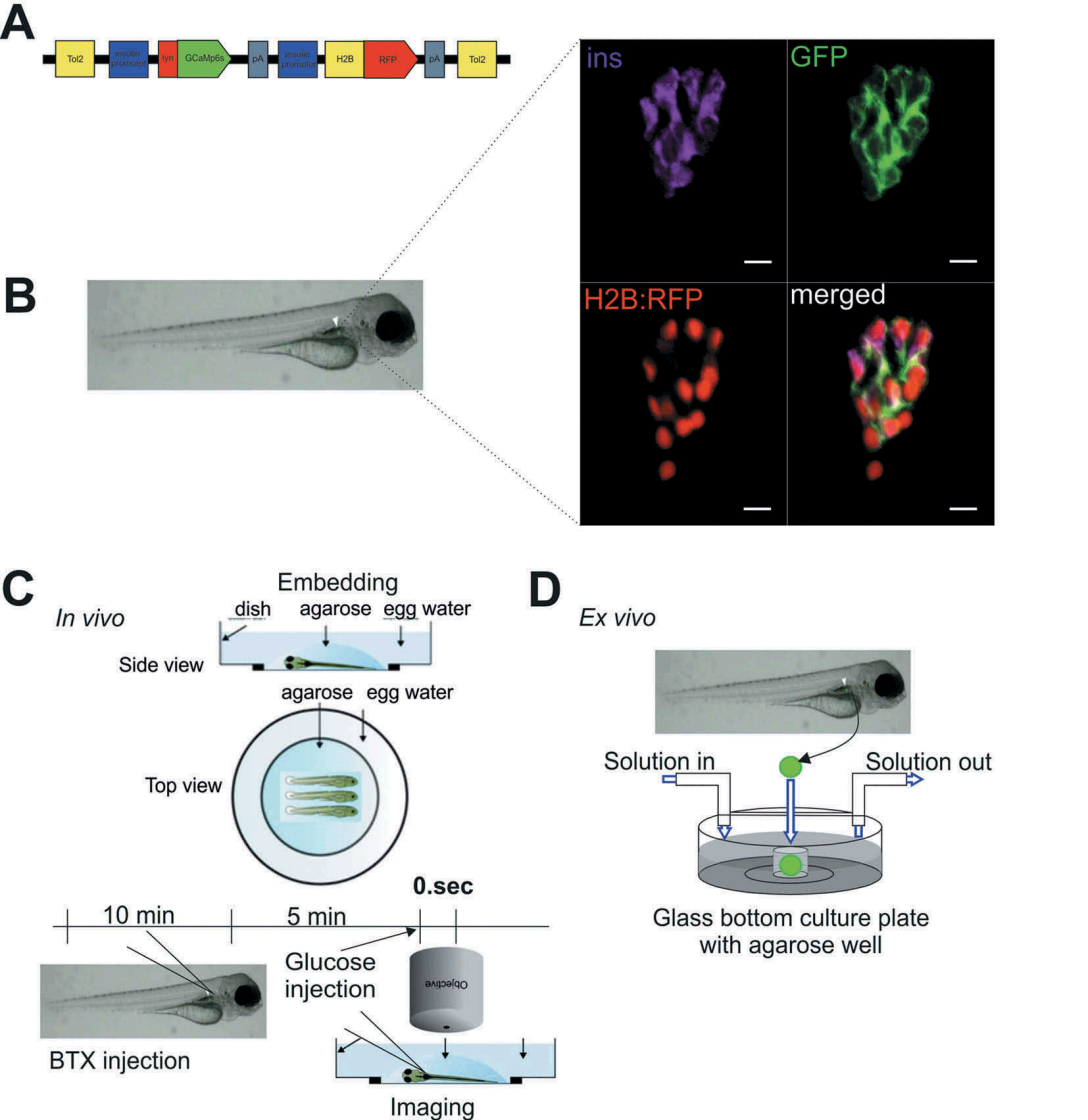
Specific expression of GCaMP6s sensor in pancreatic β-cells enables the study of Ca2+ fluxes in live zebrafish. **(A)** Illustration of the construct used to generate Tg[ins:lynGCaMP6s ins:H2BtagRFP] (ins:lynGCaMP6s). The GCaMP6s sensor was linked N-terminal with a lyn membrane tag. The additional ins:H2BtagRFP sequence was added to the construct to ease the screening of transgenic embryos and to identify the β-cells due to the nuclear specific H2BtagRFP signal. **(B)** Antibody staining on 5 days post fertilization (dpf) ins:lynGCaMP6s embryos detecting >95% overlapping of endogenous insulin and GCaMP6s (using polyconal GFP antibody) and >80% overlapping of H2B:RFP and GCaMP6s (n = 12 larvae, 20 β-cells per larva were analysed). **(C)** Schematic outline of intravenous glucose injection of zebrafish larvae. Using α-bungarotoxin (BTX) protein injection, the zebrafish larvae are paralyzed. After 10–15 min, the larvae are embedded in low melt agarose. Using a micromanipulator assembled to the microscope, glucose (in combination with Rhodamine B isothiocyanate-Dextran) is injected to the dorsal aorta of the embryo while the imaging is running. **(D)** Schematic outline of perfusion system on extracted pancreatic islet of zebrafish embryos. Islets from ins:lynGCaMP6s fish are isolated and placed into glass-bottomed culture plates with agarose gel wells containing islet media for Ca^2+^ imaging as solutions are changed. Scale bar = 10 µm

**Figure 2.**
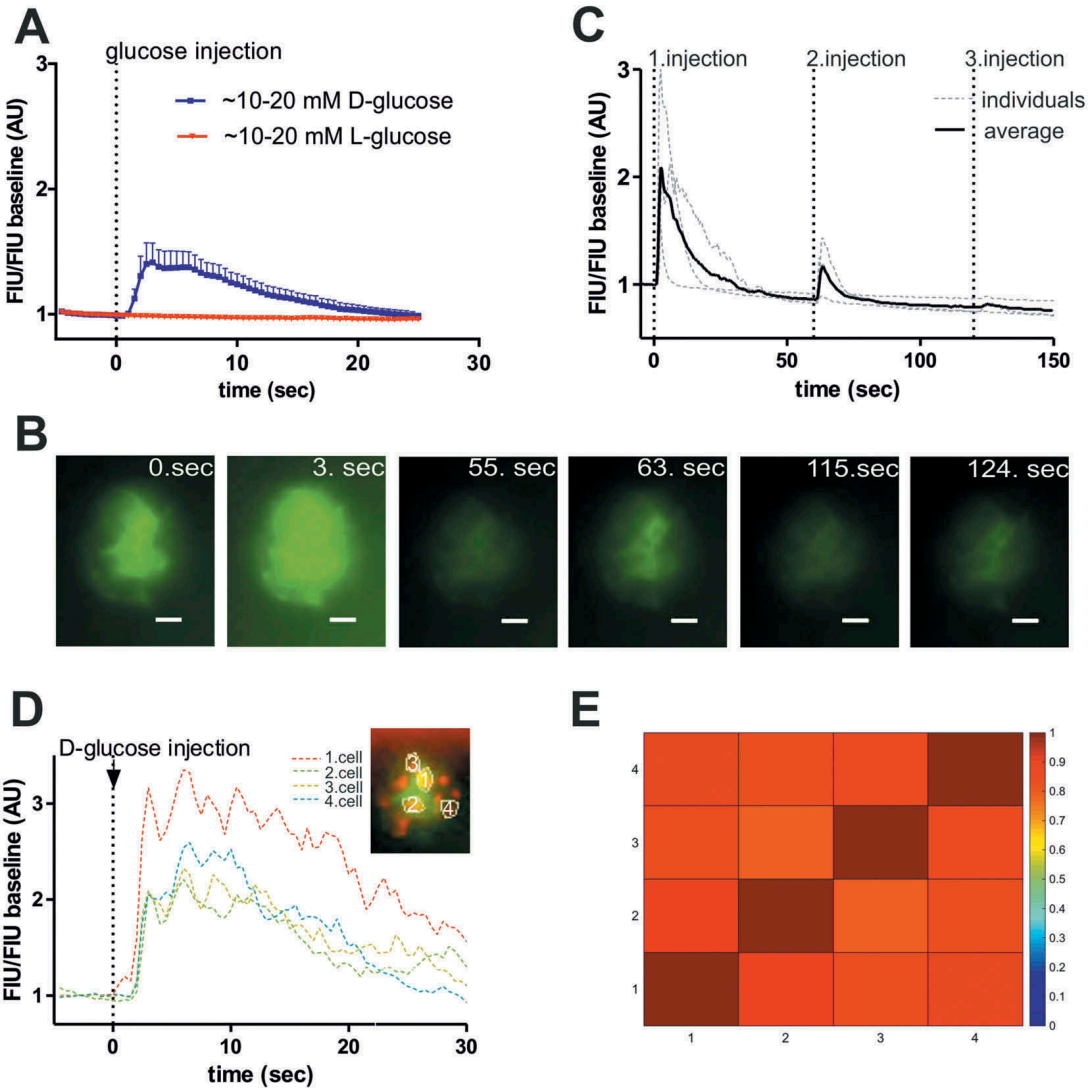
Zebrafish β-cells show rapid glucose response in vivo. **(A)** *In vivo* experiments in larva revealed rapid fluorescence signal alteration (peak within 5 sec) in response to intravenous injection of >10 mM D-glucose (blue line) (5 dpf) (n = 8 larvae). To confirm that the response is due to D-glucose metabolism, L-glucose (red line) using same concentration (>10 mM) was also injected and showed no response (n = 5 larvae). **(B)** Fluorescence image series of the islet showing β-cell specific GCaMP6 signal at the indicated time-points (sec) after intravenous injection of D-glucose (>10 mM final concentration) in 5 dpf larva. Scale bar = 10 µm **(C)** Quantitative GCaMP6 signal intensity in the larvae shown in (**B**), and two other individual larvae (gray dotted lines), showing calcium influx after sequential glucose injections (1.injection: >10 mM, 2. Injection: >20 mM, 3. Injection: >30 mM estimated final concentration). FIU intensities for the whole islet were determined using Image-J and normalized to the FIU baseline (intensity measured in the first time point). **(D)** Representative fluorescence traces of manually selected β-cell areas (n = 4, based on red RFP expression as indicated) in a 5 dpf old zebrafish larva, normalized to initial fluorescence intensity, while 1–2 nl of 0.5 M D-glucose (>10 mM final concentration) was intravenously injected *in vivo* (at dotted black line). After glucose injection β-cell areas show oscillation. **(E)** Cross-correlation matrix (determined by PeakCaller, MATLAB) of the 4, in (D) indicated, β-cell areas after intravenous glucose injection. The panel represent one islet with 4–4 cell comparison. Colour map key is given to the right of the panel. Further examples are shown in Sup. Fig. 2B. FIU = Fluorescence Intensity Units (AU, Arbitrary Units).

To enable *in vivo* studies of β-cell [Ca^2+^]_i_ in zebrafish larva, we generated a transgenic line expressing a genetically encoded membrane-tagged Ca^2+^-sensor together with a Histone-tagged RFP, both under control of the insulin promoter (Tg[ins:lynGCaMP6s; ins:H2B-RFP], referred to hereafter as ins:lynGCaMP6s; Figure 1(A,B)). The combined expression of lynGCaMP6s and H2B-RFP in β-cells enabled rapid β-cell identification and measurements of Ca^2+^ responses in selected β-cell areas. We further established protocols for determining β-cell specific Ca^2+^ changes either in the living embryo (Figure 1(C)) or in isolated islets (Figure 1(D)). In the first set of experiments, we recorded β-cell responses to different routes of glucose administration. To simulate physiologically relevant feeding-related gradual and continued increase of blood glucose level, we tested injections of glucose into the yolk, the pericardium or the hindbrain ventricle. In all approaches, the glucose injection induced Ca^2+^ fluctuation in β-cells (Sup. Fig. 1D–F), but the responses were very variable, with delays between injection and response ranging from seconds to minutes and the number of responsive cells varying from 5–30% (Sup.Fig. 1A–C). In contrast, intravenous injection of D-glucose (~10–20 mM final concentration) in wild-type 5 dpf larvae evoked rapid and robust elevations in the GCaMP6s fluorescence in almost all labelled cells (Figure 2(A), blue line). Sequential intravenous glucose injections resulted in repeated similar rapid responses, but the normalized Fluorescence Intensity Unit (FIU) peaks decreased after the second and third injection (Figure 2(B,(C)). Control injections with the same concentration of L-glucose caused no GCaMP6s signals (Figure 2(A), red line), confirming that the response is due to glucose metabolism, rather than osmotic stress or other potential injection artefacts. In intravenously D-glucose-injected embryos, the fluorescence increased almost immediately after injection, and reached its peak within 5 seconds, followed by oscillations across the islets (5 ± 1 peaks/minute; Figure 2(D), Sup.Video 1 and 2). Correlation analyses revealed that glucose-induced responses are synchronized across the islet when the glucose is injected intravenously (Figure 2(E) and Sup. Fig. 2B), which was not the case for yolk injection (Sup. Fig. 2C), indicating that the *in vivo* responsiveness of zebrafish β-cells to glucose depends on the route of glucose administration.

### Zebrafish islets are glucose responsive in early developmental stages

**Figure 3.**
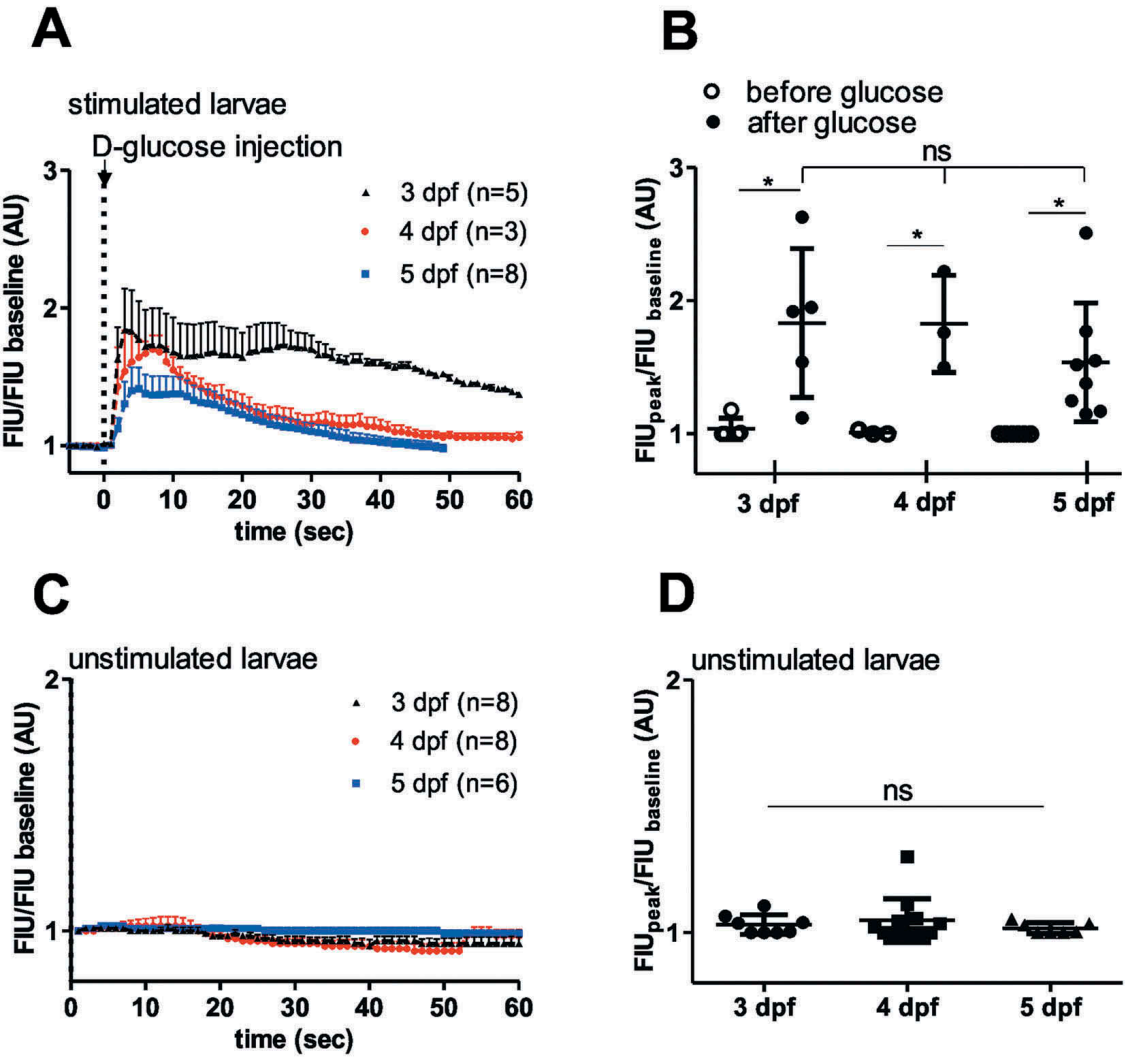
Similar *in vivo* glucose responses in 3 dpf, 4 dpf and 5 dpf zebrafish larvae. **(A)** Quantitation GCaMP6 signal intensity show rapid fluorescence signal alteration after intravenous injection of D-glucose (>10 mM final concentration) in 3, 4 and 5 dpf larvae (n = 5, 3 and 8 larvae, respectively), **(C)** and no or little alteration in unstimulated larvae (n = 8, 8 and 6 larvae, respectively). FIU intensities for the whole islet were determined using Image-J and normalized to the FIU baseline (intensity measured in the first time point) **(B)** Peak FIU intensities (FIU_peak_) of the whole islets before and after glucose injection (normalized to baseline FIU intensity measured in the first time point) (curves shown in (A) at different developmental stages (3, 4 and 5 dpf) (n = 5, 3 and 8 larvae, respectively). **(D)** Peak FIU intensities (FIU_peak_) of the whole islets in unstimulated larvae (normalized to baseline FIU intensity measured in the first time point) (curves shown in (C)) at different developmental stages (3, 4 and 5 dpf) (n = 8, 8 and 6 larvae, respectively). FIU = Fluorescence Intensity Units (AU, Arbitrary Units).Data are shown as mean values ±s.d., *p < 0.05 in one-way ANOVA, Tukey’s Multiple Comparison test to compare the glucose-injected groups (between 3, 4 and 5 dpf old larvae after glucose) in (B) and the unstimulated larvae in (D), and two-tailed, paired t-test was done to compare before and after glucose groups in (B).

We next assessed whether the glucose-responsiveness of β-cells observed in 5 dpf larvae is also seen at earlier developmental stages. Studies in rodents have established that islet maturity and function vary during development, with glucose responsiveness occurring in later stages, particularly near the weaning transition.^44^ Previous studies showed that insulin-dependent glucose homeostasis in zebrafish starts at 3 dpf.^45^ Normalized FIU intensities showed similar glucose-induced [Ca^2+^]_i_ dynamics over time (Figure 3(a)), and quantification of the GCaMP6s signal revealed significantly higher peak FIU intensities after glucose injection compared to baseline (before glucose), but no significant differences between developmental stages (3, 4 and 5 dpf) (Figure 3(b)). In addition, little or no spontaneous responses were observed in unstimulated larvae at 3–5 dpf (Figure 3(c,d)). This shows that the embryonic onset of insulin-dependent glucose homeostasis correlates with the requirement for glucose-sensing mechanisms after 3 dpf.

### Zebrafish islets are uncoupled *ex vivo*

**Figure 4.**
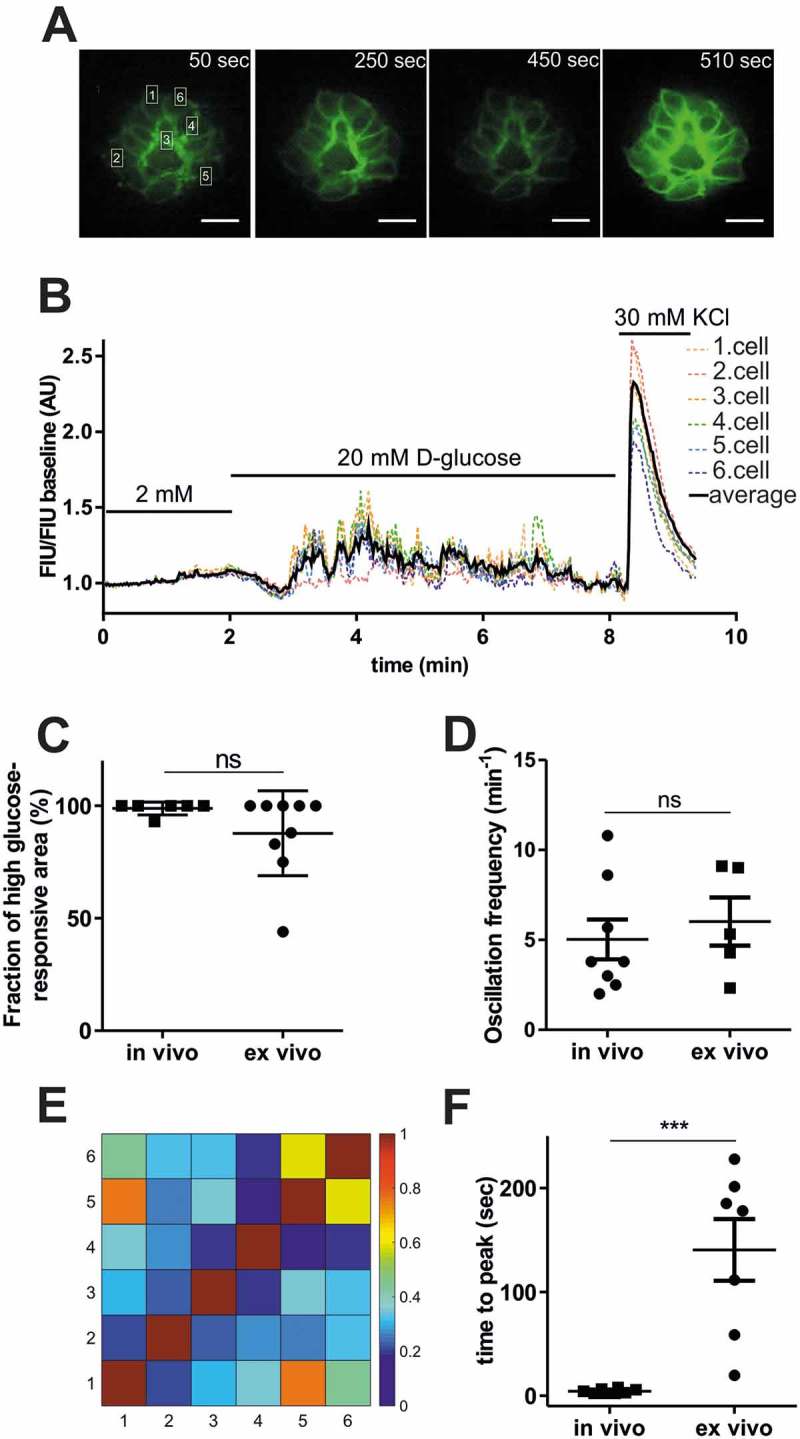
Comparison of *ex vivo* and *in vivo* glucose responsiveness. **(A)** Fluorescence image series of isolated pancreatic islet showing GCaMP6 signal alteration at the indicated time-points shown in (B) applying 2 mM, 20 mM glucose and 30 mM KCl. Single β-cell is defined as ˜3 × 5 μm white rectangular selection drawn around one piece of the GCamP6s-expressing membrane located between two individual β-cells. Scale bar = 10 µm. **(B)** Single cell GCaMP6s fluorescence traces for isolated islet from wild-type zebrafish larvae (5 dpf) after applying 2 mM, 20 mM glucose followed by 30 mM KCl. FIU intensities for the single cells (n = 6 cells) were determined using Image-J, normalized to the FIU baseline (intensities measured in the first 2 min when 2 mM glucose was applied) and displayed as FIU/FIU baseline (AU) for single cells (coloured dotted line) and for the average of the singles cells in the islet (black line). (**C)** Quantification showing the percentage of β-cells that respond upon stimulation with 20 mM glucose, *ex vivo* and the percentage of glucose-responsive β-cell area after intravenous injection of >10 mM glucose *in vivo*. (n = 8 and 9 larvae were analyzed, *in vivo* and *ex vivo*, respectively) **(D)** Average oscillation frequencies of 4 selected β-cell areas of the pancreatic islet (n = 8 islets, in each islet 4 β-cell areas were manually selected based on the red RFP expression) after intravenous injection (~1–2 nl of 0.5 M glucose, >10 mM final concentration) of 5 dpf old zebrafish larvae (*in vivo*, imaged by epifluorescence microscope), and average oscillation frequencies of individual β-cells (n = 5 islets, in each islet >6 cells were analyzed) of the isolated pancreatic islets after 20 mM glucose stimulation (*ex vivo*, imaged by spinning disc microscope). Oscillation peaks were identified by PeakCaller, MATLAB on the FIU/FIU baseline traces of individual β-cells/areas (see Sup. Fig. 2A). The peak number was divided by the time duration until oscillation was observed (˜30–60 sec, *in vivo*, ˜150–300 sec, *ex vivo*) **(E)** Cross-correlation matrix (determined by PeakCaller, MATLAB) of the islet between individual cells shown in (A) and (B), with color key to the right of the matrix graph. Numbers on the x and y axes represent individual cells that are marked in (A) and (B). **(F)** Time to peak calculated from the time point when addition of the new glucose solution was finished in isolated islets, *ex vivo* (n = 7 islets, the addition took in average ˜38 seconds), and when glucose was injected in living zebrafish larvae, *in vivo* (n = 8 islets from n = 8 larvae). Data are shown as mean values ±s.e.m., *p < 0.05 in unpaired, two-tailed t-test between *ex vivo* and *in vivo* groups in (C), (D) and (F). FIU = Fluorescence Intensity Units (AU, Arbitrary Units).

To assess the concentration-dependence of glucose and other nutrients on β-cell excitation, we performed perfusion studies with isolated pancreatic islets (*ex vivo*) from 5 dpf old zebrafish larvae (Figure 1(D)). Isolated larval islets were first incubated in 2 mM glucose solution, then stimulated with 20 mM glucose solution and at the end of the recording, 30 mM KCl solution was applied to confirm excitability. In these experiments, higher resolution imaging by spinning disc microscopy enabled single cell analysis of the isolated islets. Consistent with the *in vivo* data, 20 mM glucose increased GCaMP6s FIU/FIU baseline intensity (Figure 4(A,B)), almost all β-cells (83% ± 24%, Figure 4(C)) responded to the glucose stimulus, and the induced [Ca^2+^]_i_ oscillated with similar frequencies (5 ± 1 and 6 ± 1 peaks/minute, respectively, Figure 4(D)). However, in contrast to what was observed after intravenous glucose injection, *ex vivo* β-cell responses were out of phase (correlation analyses shown Figure 4(E,C), Sup. Fig. 3 and 2B). Notably, the time to peak in the *ex vivo* studies were highly variable (ranging from a few seconds to minutes) and on average were more than 30 times longer than those seen after intravenous glucose injection (4.4 ± 0.5 sec *in vivo* and 140 ± 30 sec *ex vivo*, Figure 4(F)).

### L-glutamine and palmitic acid also stimulate [Ca^2+^]_i_ influx in zebrafish pancreatic islet

**Figure 5.**
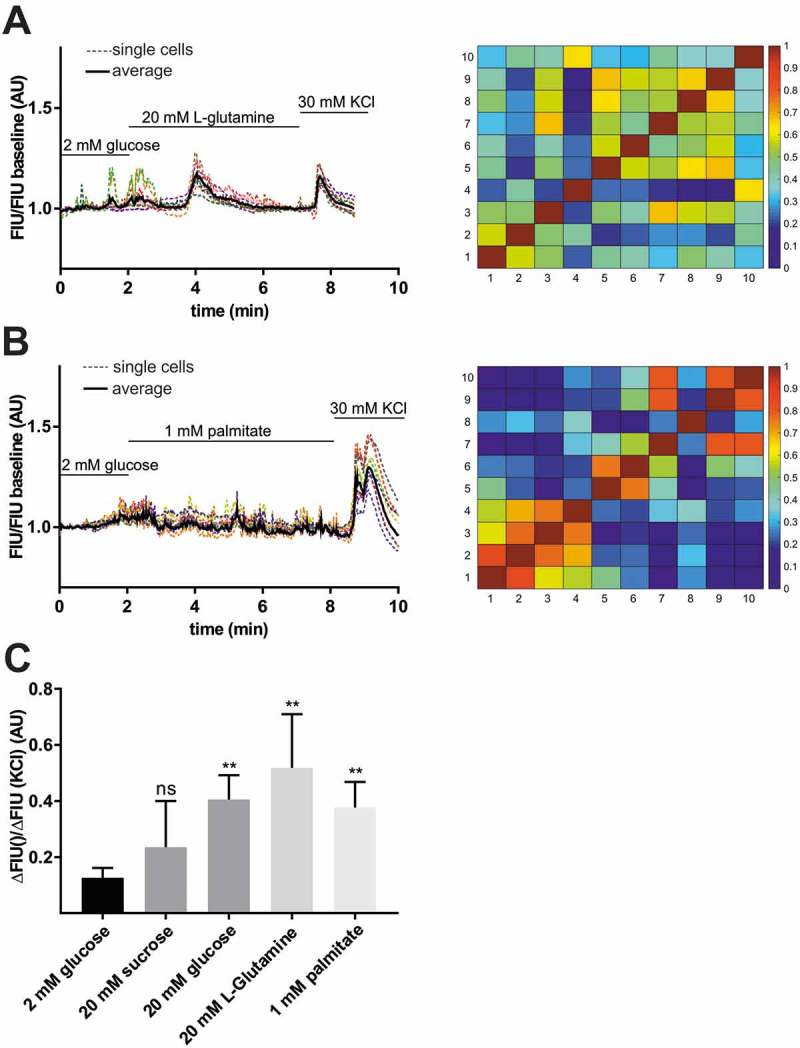
Stimulation of β-cell by L-glutamine and palmitic acid. Representative single cell GCaMP6s fluorescence traces for isolated islet from wild-type zebrafish larvae (5 dpf) after applying 2 mM glucose followed by 20 mM L-glutamine **(A)**, and 1 mM palmitic acid **(B)** stimulation and 30 mM KCl. FIU intensities for the single cells (n = 10–10 cells) were determined using Image-J, normalized to the FIU baseline (intensities measured in the first 2 min when 2 mM glucose was applied) and displayed as FIU/FIU baseline (AU) for single cells (coloured dotted line) and for the average of the singles cells in the islet (black line). Cross-correlation matrices (determined by PeakCaller, MATLAB) of the islets between individual cells shown in (A) left panel and (B) left panel, with color key to the right of the matrix graph. Numbers on the x and y axes represent individual cells. Cross-correlation analysis was done on the section where L-glutamine/palmitate stimulation was applied (excluding KCl section). **(C)** Calcium responses to 20 mM L-glutamine and 1 mM palmitic acid when compared to 2 mM glucose. The changes in GCaMP6s fluorescence intensity in β-cells of wild-type pancreatic islets were averaged for the whole islet and the FIU peak intensity upon different nutrient stimulation was normalized to the FIU peak intensity upon KCl stimulation (n = 23, 7, 5, 4 and 7 islets were analyzed for 2 mM glucose, 20 mM glucose, 20 mM L-glutamine, 20 mM sucrose, and 1 mM palmitic acid stimulation, respectively) (in each islet at least n = 5–8 cells were analyzed and the FIU peak intensity was measured in the average FIU trace for the whole islet). Data are shown as mean values ±s.e.m., *p < 0.05 in unpaired t-test between 2 mM glucose and 20 mM sucrose, 20 mM L-glutamine, and 1 mM palmitic acid stimulation. FIU = Fluorescence Intensity Units (AU, Arbitrary Units).

In mammalian β-cells, excitation occurs not only in response to glucose but also to various other stimuli. ^46–49^ To test whether amino acids and free fatty acids contribute to [Ca^2+^]_i_ signalling of zebrafish pancreatic islets, perfusion experiments with L-glutamine and palmitic acid on extracted islets were carried out. Whole islets extracted from 5 dpf zebrafish larvae showed increase in GCaMP6s FIU/FIU baseline intensity when 20 mM L-glutamine or 1 mM palmitic acid alone and no glucose was applied in the perfusion system (Figure 5(A,B)). To confirm that the responses were specific to the nutrients and were not due to osmotic stress, we performed perfusion experiments with 20 mM sucrose on the extracted pancreatic islet; no significant increase in GCaMP6s fluorescence intensity was observed. In all cases, islets were excitable, as revealed by the change in fluorescence upon 30 mM KCl stimulation (Figure 5(C)). Again, Ca^2+^ fluctuations occurred asynchronously across the whole islet in all *ex vivo* conditions, indicating that β-cells were not coupled with one other (Figure 5(A,B), right panels). These results revealed that short-term exposure to L-glutamine alone or to palmitic acid, without glucose, stimulates [Ca^2+^]_i_ influx in embryonic zebrafish pancreatic islets.

### *Ca_v_1.2/isl^m458^* mutant zebrafish are hyperglycemic

**Figure 6.**
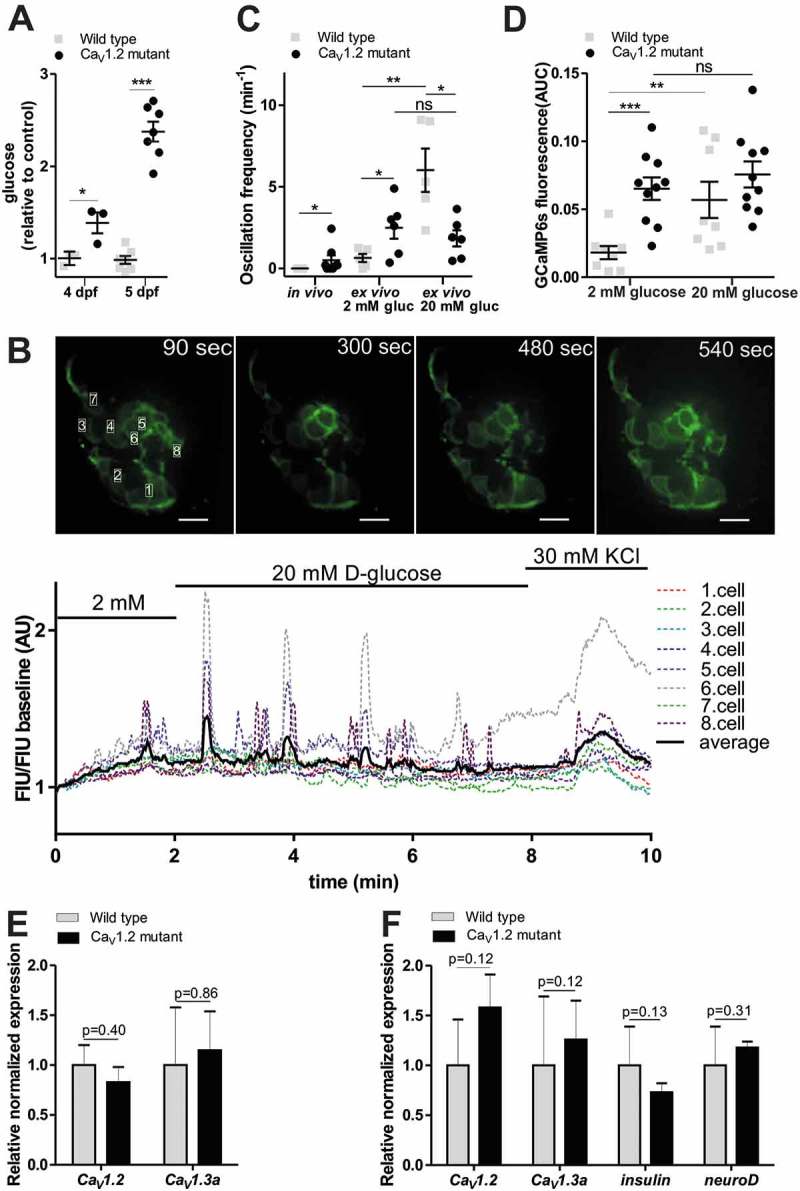
*Ex vivo* imaging of β-cells Ca^2^+_i_ dynamics in Ca_V_1.2 mutant zebrafish larvae. **(A)** *Ca_V_1.2/isl^m458^* mutant shows elevated whole larval glucose levels at 4 and 5 dpf. Glucose levels measured at 4 and 5 dpf whole larval extracts (pool of 10–10 larvae), normalized to the protein level measured by Nanodrop, and showed as relative to the average control value. (n = 4 and 8 biological replicates, respectively) (at 5 dpf, results combined from 2 independent experiments) (***p < 0.0001, two-tailed t-test). **(B)** Fluorescence image series of an isolated islet from 5 dpf old *Ca_V_1.2* mutant crossed with ins:lynGCaMP6s larva (upper panel). Quantitation of GCaMP signal intensity in the time series shown in the upper panel, indicating FIU/FIU baseline changes after 20 mM glucose as well as after 30 mM KCl stimulation (lower panel). **(C)** Quantification of GCaMP6s fluorescence intensity peaks determined by PeakCaller, MATLAB *in vivo*, without any stimulation (n = 6 wild type and 8 *Ca_V_1.2* mutant larva), and *ex vivo*, upon 2 and 20 mM glucose stimulation in wild-type and *Ca_V_1.2* mutant larva (n = 7–7 and 6–6 larvae, respectively), shown as oscillation frequency (min^−1^) (peak number/cell number*minute). β-cell number was defined based on red nuclei RFP expression in one focal plane (4–10 cells per islet were analyzed) (**p < 0.005, two-tailed, nonparametric Mann-Whitney test) **(D)** Quantification of GCaMP6s fluorescence intensity *ex vivo* in wild-type and *Ca_V_1.2* mutant larvae shown as area under curve (AUC) upon 2 mM and 20 mM glucose stimulation normalized to the treatment duration (n = 9 and 10 larvae, respectively) (***p < 0.0005, two-tailed, unpaired t-test). **(E)** Relative normalized *Ca_V_1.2* and *Ca_V_1.3a* mRNA expression levels as determined by RT-qPCR (reverse transcriptase quantitative PCR) in 5 dpf old wild type and *Ca_V_1.2* mutant larvae (pools of 15 whole larvae) (n = 3 biological replicates, 2 technical replicates) **(F)** Relative normalized *Ca_V_1.2, Ca_V_1.3a, insulin* and *neuroD* mRNA expression levels determined by RT-qPCR in isolated pancreatic islets from 5 dpf old wild type and *Ca_V_1.2* mutant larva (pools of 15 islets) (n = 3 biological replicates, 2 technical replicates). Data are shown as mean values ±s.e.m (*p < 0.05, two-tailed t-test). FIU = Fluorescence Intensity Units (AU, Arbitrary Units).

Next we used the ins:lynGCaMP6s reporter to assess β-cell specific defects in L-type calcium channel mutant zebrafish embryos. Currently, the genetic roles of *Ca_V_1.2* and *Ca_V_1*.3 in glucose homeostasis are not well defined. Physiological studies indicate that *Ca_V_1.2* and *Ca_V_1.3* are key regulators of mammalian glucose-induced insulin secretion (for a review, see ref.^43^), but corresponding mouse knockout models displayed only minor effects on insulin secretion and glucose homeostasis, perhaps due to compensation by other channels. In particular, it was found that *Ca_V_1.2* in β-cells is required for proper first-phase glucose-induced insulin secretion, and that *Ca_V_1.3* may modulate second-phase secretion, each of which is significant for normal glucose tolerance.^40,43^ The zebrafish has three *Ca_V_1.2* and *Ca_V_1.3* encoding gene orthologues, of which two – *cacna1c* (encoding *Ca_V_1.2*) and *cacna1da* (*Ca_V_1.3a*) *–* show expression in the developing pancreatic islet.^50–52^ The third gene *cacna1db* (*Ca_V_1.3b*) is exclusively expressed in the central nervous system.^51^ To test cell type specific expression of *Ca_V_1.2* and *Ca_V_1.3a*, we performed whole mount stains in different endocrine GFP-reporter stains (Sup. Fig. 4). We found that *Ca_V_1.2* and *Ca_V_1.3a* are expressed in all hormone-positive pancreatic cells of embryos older than one day (Sup. Fig. 4.). To determine requirements of these genes in glucose homeostasis, we took advantage of previously described mutants for *Ca_V_1.2 (isl, isl^m485^, island beat)*^50,53^ and *Ca_V_1.3a* (*gem, gem*^tc323d^, *gemini*).^54,55^ Both mutant alleles carry premature stop codons that result in the expression of truncated (and most likely inactive) proteins. Measurements of free glucose in control and mutant embryos showed elevated glucose levels in 4–5 dpf *Ca_V_1.2/isl^m458^* (Figure 6(A) and Sup. Fig. 5) but normal glucose levels in 5 dpf *Ca_V_1.3a/gem*^tc323d^ mutants (Sup. Fig. 6). These data suggest impaired β-cell functionality in the *Ca_V_1.2/isl^m458^* mutant fish.

To test whether *Ca_V_1.2/isl^m458^* mutants affect β-cell specific [Ca^2+^]_i_ dynamics, the mutation was crossed into the ins:lynGCaMP6s background. *In vivo* fluorescence recordings of untreated 5 dpf transgenic larvae showed significantly higher basal islet [Ca^2+^]_i_ dynamics in mutants than control animals (Figure 6(C), Sup. Video 3 and 4). Hyperglycemia in these animals raised the possibility that non-islet effects could underlie the increased [Ca^2+^]_i_ dynamics in the *Ca_V_1.2/isl^m458^* mutants. To eliminate non-islet effects, we performed *ex vivo* recordings of isolated mutant and control islets. Importantly, increased [Ca^2+^]_i_ dynamics in mutants were maintained in isolated islets and appeared to be glucose independent. In particular, we found that in mutant islets the change from 2 mM to 20 mM glucose had no significant effect on the peak frequency (2 mM: 0.6 ± 0.3 and 2.6 ± 0.7 peaks/min; 20 mM: 6.0 ± 1.0 and 1.8 ± 0.5, control and mutant, respectively) (Figure 6(C)), or on the integrated signal intensity (2 mM: 0.018 ± 0.004 and 0.065 ± 0.008 AUC; 20 mM: 0.057 ± 0.013 and 0.074 ± 0.011, control and mutant, respectively) (Figure 6(D) and Sup. Fig 7A), while in control islets these parameters were significantly increased by increasing glucose levels. Thus, there is a counterintuitive increase in [Ca^2+^]_i_ oscillations at basal glucose in the mutant islets. In mouse, *Ca_V_1.3* subunit knockout was found to be compensated by *Ca_V_1.2* upregulation.^42^ To test whether upregulation of *Ca_V_1.3a* may account for the *Ca_V_1.2/isl^m458^ m*utant phenotype, we performed RT-qPCR (reverse transcriptase quantitative PCR) analyses. However, transcripts levels for *Ca_V_1.2* and *Ca_V_1.3a* in whole embryos (Figure 6(E)) and isolated islets (Figure 6(F)) showed no significant differences between controls and mutants. These data show that genetic loss of *Ca_V_1.2* results in glucose-independent hyperactivation of β-cells, and suggest that this activation is independent of cell-autonomous compensatory effects.

## Discussion

We have successfully assessed [Ca^2+^]_i_ dynamics in β-cells of zebrafish pancreatic islets, both *in vivo* after injection of glucose and *ex vivo* using perfused isolated islet. We found that zebrafish β-cells are directly glucose-responsive, and that responsiveness is established early in embryonic development. These data are consistent with a conserved glucose sensing and stimulus-secretion coupling mechanism in fish and mammals. However, our analyses also showed that glucose-induced β-cell responses in perfused embryonic zebrafish islets are not well-synchronized, while β-cells in perfused mammalian islets show well-synchronized [Ca^2+^]_i_ oscillations throughout the whole islet.^56–58^ In agreement with very recent *ex vivo* Ca^2+^ recordings of perfused juvenile zebrafish islet,^39^ the data suggest that zebrafish islets are uncoupled, at least in the developing zebrafish.

We also evaluated different approaches for glucose administration in living zebrafish embryos. It is important to note that our *in vivo* imaging data, in contrast to the *ex vivo* data, did not allow analyses at single cell resolution. The combination of the deep tissue localization, the frequent presence of light absorbing pigment cells, and permanent slow tissue movements due to gut peristaltic movement prevented more detailed analyses. However, our data revealed that embryonic *in vivo* β-cell responses are immediate and synchronized when glucose is directly injected into the bloodstream, and that gradually increasing blood glucose levels result in delayed, non-synchronized [Ca^2+^]_i_ fluctuations, very similar to those found in glucose-stimulated perfused islets. Currently, we can only speculate about the mechanism underlying the synchronized responses after intravenous glucose injection. Possibly, the synchronicity is caused by a cell-autonomous but simultaneous response of all β-cells to the simultaneous prompt exposure to a high glucose challenge. Alternatively, synchronicity could be triggered by a currently undefined paracrine signal. In this context, the much faster [Ca^2+^]_i_ dynamics in living animals, as compared to isolated islets, could indicate the involvement of neuronal driven triggers. Consistent with this possibility, it was recently shown that the embryonic zebrafish islet is innervated at the relevant time points, and that a neurotransmitter, namely galanin expressed in these neurons, is involved in blood glucose regulation.^59,60^ Notably, the delayed glucose response in the isolated zebrafish islets is similar to that seen in mammalian islets. While this delay is attributed to the time taken for glucose diffusion and metabolism,^61,62^ it might be further affected by the cleavage of neural connection during the islet isolation procedure.^60^ Measurements of real-time glucose response of pancreatic mouse islets by autofluorescence NAD(P)H signals also suggests that there might be differences in glucose metabolism after isolation.^63^ However, the time to reach the initial GCaMP fluorescence peak after glucose injection in mice *in vivo* (~1–2 minutes) is still much slower than in our study in zebrafish (˜5 sec).^35^ Therefore, we also cannot exclude the possibility that the fast response in zebrafish is caused by bypassing glucose metabolism, with glucose activating [Ca^2+^]_i_ by other mechanisms, such as the swelling-sensing LRRC8 channels, for example, similar to mouse β-cells.^64^ Detailed analyses is required to investigate the potential function of the LRRC8 proteins in zebrafish β-cells, but expression profiling of zebrafish islets cells suggests that the 5 *lrrc8* genes are differentially expressed between these cell types.^52,65^

Our finding that both palmitate and L-glutamine resulted in elevated [Ca^2+^]_i_ correlates with increased Ca^2+^ signalling and insulin secretion in mammalian islets exposed to free fatty acids and amino acids. However, and in contrast to mammalian islets, zebrafish embryonic islets did not require the simultaneous presence of glucose for elevated [Ca^2+^]_i_ signals.^47,66,67^

Our studies further reveal a novel central role of *Ca_V_1.2* in β-cell specific stimulus-secretion coupling in zebrafish. We find that genetic loss of *Ca_V_1.2* but not of *Ca_V_1.3a*, results in elevated glucose level during development. Further, we show that β-cells in *Ca_V_1.2/isl^m458^* mutant zebrafish display increased basal [Ca^2+^]_i_ and a complete loss of glucose-responsiveness. Future studies will be required to unravel the underlying mechanism of the increased basal activity of the *Ca_V_1.2* truncated β-cells. The counterintuitive increase in [Ca^2+^]_i_ oscillations at basal glucose in the mutant cells could be caused by an overcompensation of other VDCCs, as was seen in *Ca_V_1.3* knockout mice.^42^ While our qPCR analyses revealed no evidence for up-regulation of *Ca_V_1.2* and *Ca_V_1.3a*, this does not exclude the possibility of dysregulation of other Ca^2+^ channels (N-, P/Q-type), that might be expressed and functional in the zebrafish islet.^52^ Importantly, our studies also suggest that β-cell specific *Ca_V_1.2* functions might be very different in zebrafish and mice. Notably, *Ca_V_1.2* mutant mice are not viable and therefore comparable embryonic β-cell defects have not been determined in the mouse mutants.^68^ However, β-cell specific loss of *Ca_V_1.2* has only moderate effects on [Ca^2+^]_i_ handling and glucose responsiveness in mice, despite significantly reduced Ca_V_ currents and inhibition of first-phase insulin secretion.^40^ The expression of *Ca_V_1.2* not only in β-cells but rather in all pancreatic endocrine cells leaves other possibilities for non-cell autonomous β-cell defects due to paracrine effects such as dysfunctional α- or δ-cells. A disturbed intra-islet communication (for a review see ref.^69^) could explain why β-cell phenotypes in *Ca_V_1.2/isl^m458^* mutants appear to be completely different from those seen in β-cell specific *Ca_V_1.2* knock-out mice.

Our demonstration of glucose-dependence of [Ca^2+^]_i_ provides support for zebrafish as an appropriate model to study stimulus-secretion coupling in the pancreas, both for fundamental understanding of secretion control, and for development of novel approaches to β-cell related diseases. However, in defining distinct consequences of manipulation of the voltage-gated L-type Ca^2+^ channel, our work also highlights the need to determine underlying differences between mammalian and zebrafish β-cell physiology to better understand this framework for applying zebrafish as a diabetes model.

## Materials and methods

### Zebrafish breeding and maintenance

Zebrafish (*Danio rerio*) were maintained according to standard protocols.^70^ Developing embryos were staged by hours post fertilization (hpf) when incubated at 28°C.^71^ All procedures were approved by the Austrian Bundesministerium für Wissenchaſt und Forschung (GZ BMWFW-66.008/0004-WF/II/3b/2014).

### Construction of Tg[ins:lynGCaMP6s,ins:H2B:RFP]

The lyn membrane tag sequence was PCR-amplified from pcDNA8D3cpv (Addgene plasmid #36323; Primer1: 5′TGGGATCCCCACCATGGGCTGCATCA-3′; Primer 2: 5′-TCTCTAGACCGGTATCGGGTGGATCGATTCTCAGTTTGGGTGGGGTCTTCATGTCCACGC-3′) and ligated into the pME vector using the BamHI and XbaI sites. Subsequently, the GCaMP6s sequence from Tol2 HuC GCaMP6s vector were added using Age1 sites for ligation. The final construct was assembled by 4-fragment Gateway cloning (LR Clonase II Enzyme mix; Invitrogen, 11–791-100) using pDestTol2CG2 ins:H2B-RFP (Addgene plasmid), p5E insulin promoter, p3E polyA and the pME lynGCaMP6s plasmids.

### Immunohistochemistry

Immunostaining of whole embryos or larvae was performed as previously described.^72^ Larvae were collected at 5 dpf, and fixed for 2 hours at room temperature in 4% PFA/1% DMSO in PBS, then washed 3 × 5 minutes with 1 × PBS/0.1% Tween. Zebrafish embryos were incubated for one hour in blocking solution (PBS/1%DMSO/1%BSA/1% Triton), then incubated overnight at 4°C with primary antibodies diluted in blocking solution. Afterwards, washing was conducted with PBS+0.2% Triton. Primary antibodies and dilutions were: guinea pig anti-insulin (1:200, Dako, A0564), rabbit anti-GFP (1:200, Torrey Pines Biolabs, NC9589665). Secondary antibodies (1:1000 dilution) were Alexa-conjugated (Invitrogen). Embryos were washed and then reblocked, and incubated in secondary antibody overnight at 4°C.

### Whole-mount in situ hybridization (WISH)

WISH was performed as described previously.^73^ Antisense probes were Digoxigenin (DIG RNA Labeling Mix, Roche, 11277073910) labelled and visualized with α-Digoxigenin-AP antibody (1:4000, Roche, 11093274910). The following probes were used:

Ca_V_1.2: Ca_V_1.2 Xba S: GGACAACTTTGACTATTTAACACGG; Ca_V_1.2_R: CTGTTCCCCCTACAAACTCG

Ca_V_1.3a: Ca_V_1.3a 5′ S: CTGAAAGGAAAGACCTCATCAAAGAG, Ca_V_13a_R: ACCAGGTGCTATAAAGTGGTAAT

All antisense RNA probes were generated by linearization of the corresponding plasmids and transcription was performed using T3, T7 or SP6 RNA polymerase. The eGFP of transgenic animals was visualized by immunostaining after the *in situ* hybridisation. Primary antibody: anti-GFP rabbit (1:200, Abcam, ab6556); secondary antibody: Alexa Fluor 488 goat anti-rabbit (1:1000, Roche, A32731). Immunostainings were performed as described above.

### *In vivo* glucose injection and imaging

Larvae (3, 4 and 5 dpf) for live imaging were anesthetized using cold-shock (embryos in egg water-containing dishes are set directly on ice) and immobilized by injection of ~10 nl of 1 mg/ml purified α-bungarotoxin (BTX, B137, Sigma-Aldrich, T9641) either to the yolk or to the gut. After 10–15 minutes, embryos were embedded in 1.2 % low melt agarose and imaged either on a Leica DM6000B microscope equipped with a SPOT-RT3 digital camera (Diagnostic Instruments, Inc., Sterling Heights, MI) (intravenous injection) or on a Zeiss Cell Observer SD using a 25X water immersion objective (brain, yolk, pericardium injection). Time lapse images were collected with 500 ms–1000 ms time intervals, 50–100 ms exposure for GCaMP6s, and 150 ms for RFP, 2 × 2 binning. For adjusting the needle (GB120F-10, 69 × 1,20 × 100 mm with filament, Science Products GmbH) into the dorsal aorta, or into the hindbrain ventricle, yolk and pericardium, 5X or 10X dry objectives, and for intravenous injection of ~1–2 nl of 0.5 M glucose (Carl Roth GmBH) (final concentration of ~10–20 mM, assuming the blood volume of zebrafish embryo is 60 nl) (79) in combination with 1.25% Rhodamine B isothiocyanate-Dextran (R9379, Sigma-Aldrich) to confirm intravenous injection, 40X water objective was used (Sup.Video 1). *In vivo* imaging of hindbrain ventricle, yolk, pericardium glucose injection (˜9 nl of 1 M glucose in combination with 1.25% Rhodamine B isothiocyanate-Dextran, final concentration of ˜30 mM, assuming the total volume of a 5 dpf old larva is 300 nl),^74^ unstimulated larvae and *Ca_V_1.2/isl* mutants crossed with ins:lynGCaMP6s was carried out with a Zeiss Cell Observer SD spinning disc microscope using a 25X water immersion objective.

### Larvae islet isolation

Zebrafish embryos at 5 dpf were euthanized using cold-shock, rolled onto their left sides and, using 26 G sterile hypodermic-needles, yolk and visceral organs were removed by applying gentle pressure using needles until fully separated. The islets were identified and confirmed by either GCaMP6s green fluorescence or RFP red fluorescence. Not all of the islet surrounding tissue was removed in order to avoid the collapse of the islet. Freshly dissected islets were transferred by glass pipette to RPMI (ThermoFisher 11875–093) supplemented with 1 mM HEPES, antibiotic solution (Sigma A5955, 10 mll−1 solution), 10% fetal bovine serum and diluted with glucose-free RPMI to final glucose concentration of 6.67 mM (zebrafish islet media) in a 12-wells culture plate. Islets were incubated at 28°C for 1.5–2 hours before imaging.

### Glucose level measurement

Glucose levels in whole larvae were measured using Glucose Assay Kit (BioVision, K606-100) as previously described.^75^ In brief, pools of 10 whole larvae were collected in 200 µl cold PBS, and lysed by glass bead (0.5 mm) homogenization in Precellys24 bead homogenizer (one cycle of 20 s at 5000 rpm) (Peqlab). After centrifugation (10, 000 rpm for 30 s at 4°C), supernatants were transferred into fresh tubes, and glucose assay was carried out according to manufacturer’s recommendation. In all experiments, at least 3 biological replicates (10 whole larvae), and 2 technical replicates were measured.

### *Ex vivo* imaging

Islets from ins:lynGCaMP6s were placed into glass-bottomed culture plates containing 2 mM glucose solution (2 mM glucose in KRBH salt solution: 114 mM NaCl, 4.7 mM KCl, 1.16 mM MgSO_4_ heptahydrate, 1.2 mM KH_2_PO_4_, 2.5 mM CaCl_2_ dihydrate, 5 mM NaHCO_3_, 20 mM HEPES and 0.1 g/100 ml BSA) with agarose gel wells. Islets were imaged on a Zeiss Cell Observer SD using a 40X water immersion objective (time lapse with 500 ms-1 s time interval, 50 ms exposure for GCaMP6s) while indicated solutions were changed using a handmade perfusion system (5 ml syringes connected to plastic tubes through 26 G needles). Salts and glucose were purchased from either Roth GmbH or AppliChem GmbH. Solutions containing 2 mM and 20 mM glucose, 20 mM L-glutamine (Thermo Fischer, 25030081), 1 mM palmitic acid (sodium salt, 98%, Thermo Fischer, 416700050) and 30 mM KCl were used.

### Image analysis

Visiview soſtware (Visitron Systems, Puchheim, Germany) (for *in vivo* intravenous glucose injection experiments) and ZEN software (for *ex vivo* experiments and *in vivo* brain, yolk, pericardium injection) were used to capture time lapses, and fluorescence images were processed using ImageJ (Fiji) ROI Analyzer.^76^ StackReg plugin in the ImageJ/Fiji was used for the recursive alignment of time series, as previously described.^77^ The ROI manager in ImageJ/Fiji was used to manually draw either rectangular selection (~3 × 5 μm) around one piece of the GCaMP6s-expressing membranes located between two individual β-cells (referred as single β-cell, in *ex vivo* experiments done by Zeiss spinning disc microscopy), or polygon selections around the whole islet or 4 non-overlapping single-cell sized β-cell areas per islet that were defined based on red nuclei RFP expression (called as β-cell area) (*in vivo* experiments done by Leica epifluorescence microscopy) to measure the mean GCaMP6s fluorescence intensity (FIU) for each time frame. The mean GCaMP6s FIU per β-cell/β-cell area or per islet was normalized to the baseline (which is calculated as the mean intensity during incubation in 2 mM glucose for *ex vivo* experiments, and as the intensity in the first time point for *in vivo* experiments) and displayed as FIU/FIU baseline trace (Arbitrary Unit, AU) over time (sec or min). To correct for fluorescence decay due to photo bleaching, non-linear regression analysis (2 phase exponential decay, without weight data points and fixing of parameters) was carried out using GraphPad Prism version 5.00 for Windows (GraphPad Software, La Jolla, California USA, www.graphpad.com). Total β-cell [Ca^2+^]_i_ dynamics (*ex vivo*) was quantified by two different methods, either by calculating the area under the curve (AUC) as described previously,^35^ or for quantitative curves in Figure 5(C) and Sup. Fig. 7, the glucose and other nutrient (sucrose, L-glutamine, palmitic acid) responses are shown normalized to the change in fluorescence in response to KCl (showing maximum islet depolarization), because the maximum excitability of an islet or β-cell can vary, and the intensity of the islet’s fluorescence can also vary. For AUC analysis, we calculated the average of the single FIU/FIU baseline traces for 2 min before stimulation (basal [Ca^2+^]_i_ dynamics), and 6 min after 20 mM glucose stimulation (stimulated [Ca^2+^]_i_ dynamics), and normalized to the treatment duration (Figure 6(D)). For calculating the fraction of high glucose-responsive area (Figure 4(C)), the Ca^2+^ influx response was defined as positive when the normalized GCaMP6s FIU within the selected β-cell area (*in vivo*) or single β-cell (*ex vivo*) showed >7% increase in comparison to the time point before glucose injection or 20 mM glucose stimulation, and this increase in fluorescence intensity was higher than the peak fluorescence intensity at baseline. In all *ex vivo* experiments, β-cells that failed to show fluorescence increase (>7% compared to time point before stimulation) upon KCl stimulation were excluded from quantification. Cross-correlation analysis (Fig. 4(E), 5(A,B), Sup. Fig. 2B, 3) and identification of the peak numbers for oscillation frequencies (Figures 4(D) and 6(C)) on the FIU/FIU baseline traces of individual β-cells/areas were done by PeakCaller in MATLAB^78^ (required rise: 1%, required fall: 3%, max. lookback: 5 pts, max. lookahead: 5 pts) Cross-correlation analysis was done on the section where glucose stimulation was applied (excluding KCl section). Oscillation frequencies (min^−1^) are average peak number per minute of selected β-cells (>6 cells, *ex vivo*) or β-cell areas (4 non-overlapping single-cell sized selected region based on RFP red expression, *in vivo*) normalized to the time duration until oscillation was observed in the time series (˜30–60 sec, *in vivo*, ˜150–300 sec, *ex vivo).*

### qPCR

Total RNA was prepared from dissected islets of 5 dpf old zebrafish larvae (pool of 15 islets) and from whole zebrafish larvae (pool of 15 larvae) using Trizol (Ambion), cDNA was prepared using the First Strand cDNA synthesis kit (Thermo Scientific). qPCR was performed in a CFX Connect Real-Time System (BioRad) using the HOT Fire-Pol EvaGreen qPCR Mic Plus (Solis BioDyne). Data shown is the average of 3 biological replicates, presented as gene expression level relative to controls, after normalization to the housekeeping gene *ef1alpha*. PCR primer pairs for *Ca_V_1.2* (5′ to 3′): TCTTCAGGTGTGCGACAGGA (F), CTTCTCACAAGGGCGGTTTG (R), *Ca_V_1.3a*: CCTCAGGCTGTGCTTCTGCT (F), CACAGCTTGCCTGGCATACA (R), *insulin*: GCCCAACAGGCTTCTTCTACAAC (F), GCAGATTTAGGAGGAAGGAAACCC (R), *neuroD*: CTTTCAACACACCCTAGAGTTCCG (F), GCATCATGCTTTCCTCGCTGTATG (R), *ef1alpha*: TCTCTACCTACCCTCCTCTTGGTC (F), TTGGTCTTGGCAGCCTTCTGTG (R).

### Statistics

Graphical and statistical analyses were performed using GraphPad Prism version 5.00 for Windows, GraphPad Software, La Jolla, California USA, www.graphpad.com. Significance was tested using two-tailed distribution and either t-test or ONE-way ANOVA analysis with p < 0.05 considered significant. Data presented are generally representative of at least three independent experiments (except as indicated). Error bars represent standard error (s.e.m) (except as indicated).

## Disclosure of potential conflicts of interest

No potential conflicts of interest were disclosed.
