# Supplementary material for: In vivo monitoring of intracellular Ca2+ dynamics in the pancreatic β-cells of zebrafish embryos
Source: Islets. 2018 Dec 6;10(6):221–38. doi: 10.1080/19382014.2018.1540234 (PMC6300091; doi:10.1080/19382014.2018.1540234)
Supplement: Supplemental Material [file kisl-10-06-1540234-s001.zip › KISL_A_1540234_Figure s1-s7.docx]

**Supplementary Figure 1:** ***In vivo* glucose injection to the hindbrain ventricle, yolk, pericardium (A)** Time to peak (sec) calculated from the time point when ˜9 nl of 1 M D-glucose (˜30 mM end concentration if volume of larva is defined as 300 nl) was injected to the hindbrain ventricle (brain), the yolk, the pericardium (pericard) or to the dorsal aorta (intravenous) of the 5 dpf old zebrafish larvae (n=6, 7, 3 and 7 larvae, respectively) (unpaired two-tailed t-test, *p < 0.05). **(B)** Quantification showing the mean percentage of glucose responsive β-cell areas and **(C)** the peak FIU intensities in the whole islet (FIU_peak_) normalized to baseline FIU intensity (FIU_baseline_) *in vivo* before and after glucose injection (˜30 mM end concentration) to the hindbrain ventricle (brain), to the yolk, to the pericardium (pericard), or to the dorsal aorta (intravenous) of the 5 dpf old zebrafish larvae (n=6, 7, 3 and 7 larvae, respectively) (paired two-tailed t-test, ****p < 0.0001). β-cell areas were defined based on red nuclei RFP expression in one focal plane. A representative β-cell area is marked with a green polygon selection in (A), right panel. Peak FIU intensities were measured in the whole islet (a representative whole islet is marked as a yellow polygon selection in (A), right panel) at baseline (before glucose injection, imaged for 2 minutes) and after glucose injection (imaged for 8 minutes). Representative fluorescence traces of whole islets in individual 5 dpf old zebrafish larvae normalized to initial fluorescence intensity, while ˜9 nl of 1 M D-glucose (˜30 mM end concentration) was injected *in vivo* (at dotted black line) to **(D**) the hindbrain ventricle (brain), **(E)** the yolk, and **(F)** the pericardium (pericard). Representative brain (D, right panel), yolk (E, right panel) and pericardium (F, right panel) glucose injection in combination with Rhodamine B isothiocyanate-Dextran (red dye) to confirm the injection, lateral view. Data are shown as mean values ± s.e.m. Scale bars, 10 and 100 μm in (A) and (D)-(F), respectively.


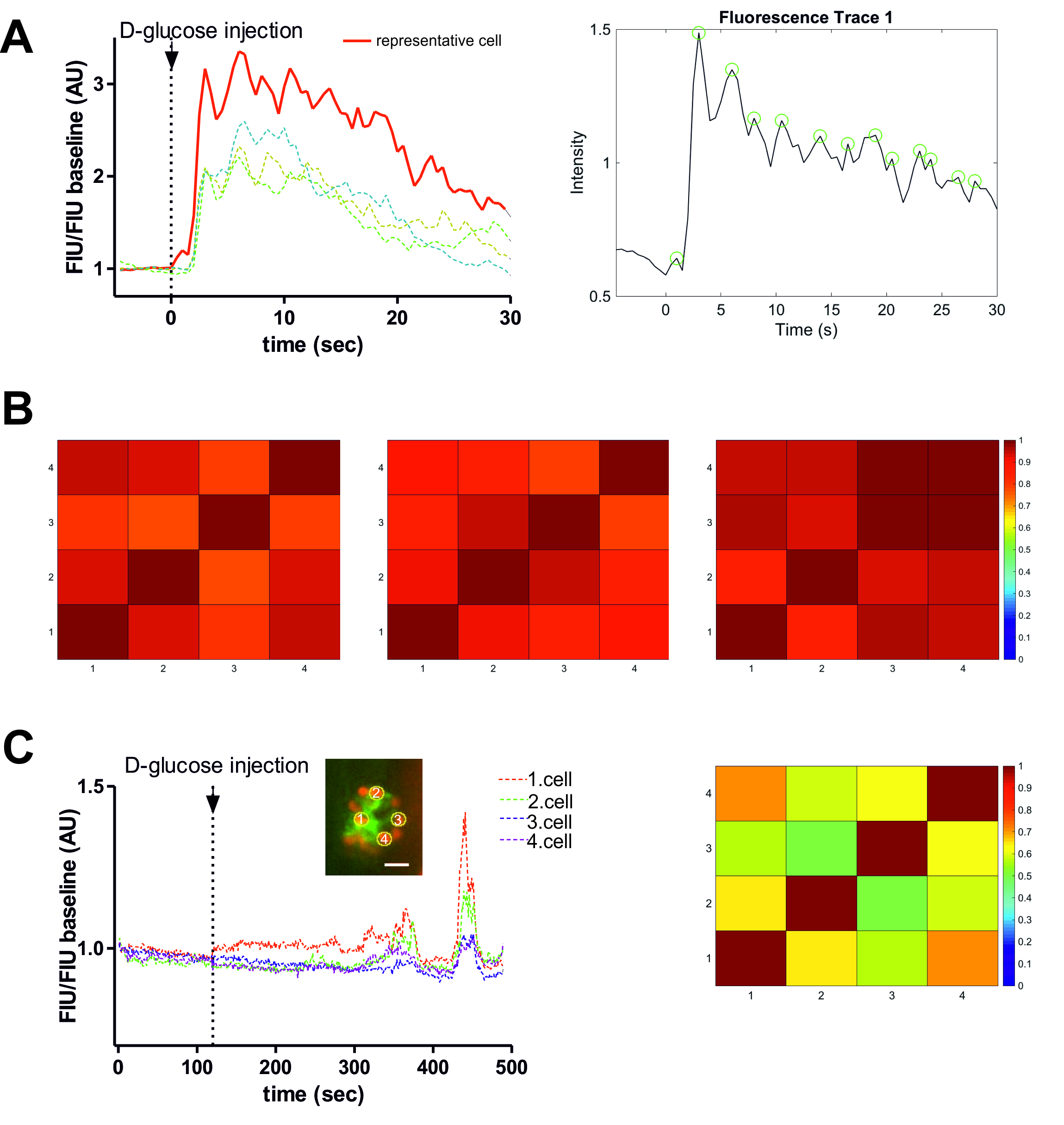


**Supplementary Figure 2: Cross-correlation analyses of pancreatic β-cell areas after intravenous and intraperitoneal (yolk) injection (A)** Representative fluorescence traces of manually selected β-cell areas (n=4, based on red RFP expression) in a 5 dpf old zebrafish larva, normalized to initial fluorescence intensity, while 1-2 nl of 0.5 M D-glucose (~10-20 mM final concentration) was intravenously injected *in vivo* (at dotted black line) (left panel). Analysis of a representative Ca^2+^ imaging data output by PeakCaller. Green circles represent PeakCaller identified peaks. Parameters were chosen as described in Materials and Methods (right panel).  **(B)** Cross-correlation matrices (determined by PeakCaller, MATLAB) of 4 selected β-cell areas of 3 pancreatic islets from 5 dpf old living zebrafish larvae after intravenous injection (~1-2 nl of 0.5 M glucose, final concentration of ~10-20 mM). Each panel represents one islet with n=4-4 cell comparison. Colour map key is given to the right of the panel. **(C)** Representative fluorescence traces of manually selected β-cell areas (n=4, based on red RFP expression) in a 5 dpf old zebrafish larva normalized to initial fluorescence intensity, while ˜9 nl of 1 M D-glucose (˜30 mM end concentration) was injected intraperitoneal to the yolk *in vivo* (at dotted black line). Cross-correlation matrix (determined by PeakCaller, MATLAB) of the 4 selected β-cell areas shown above the graph, showing lower correlation in comparison to intravenous glucose injection in (B) (C, right panel). Scale bar indicates 10 μm.


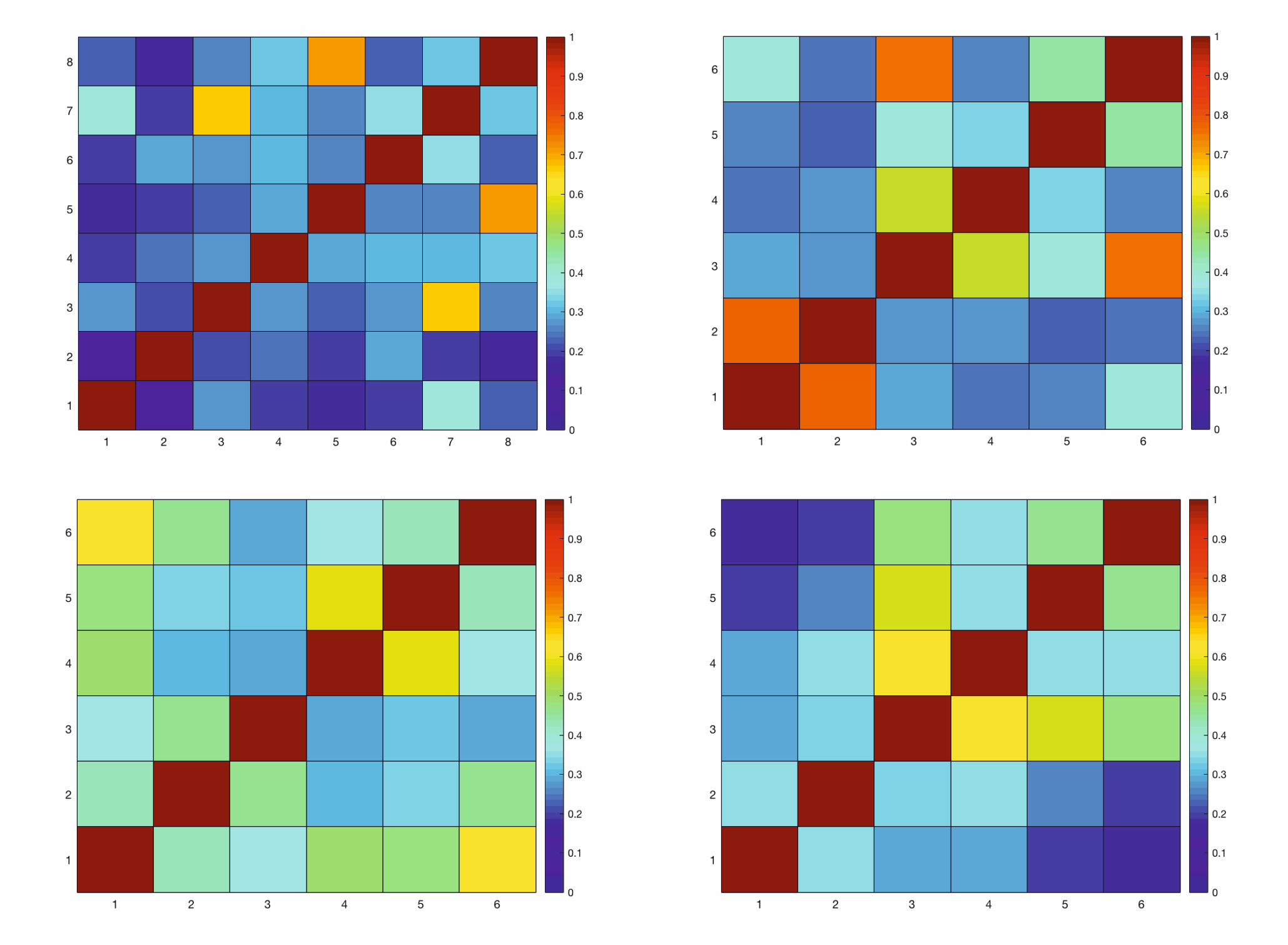


**Supplementary Figure 3: Cross-correlation analyses of isolated islets from 5 dpf old zebrafish larvae.** Cross-correlation matrices (determined by PeakCaller, MATLAB) of the β-cells from the isolated islets of 5 dpf old zebrafish larvae. Each panel represents one isolated islet with n=6-8 cell comparison. Colour map key is given to the right of the panel.


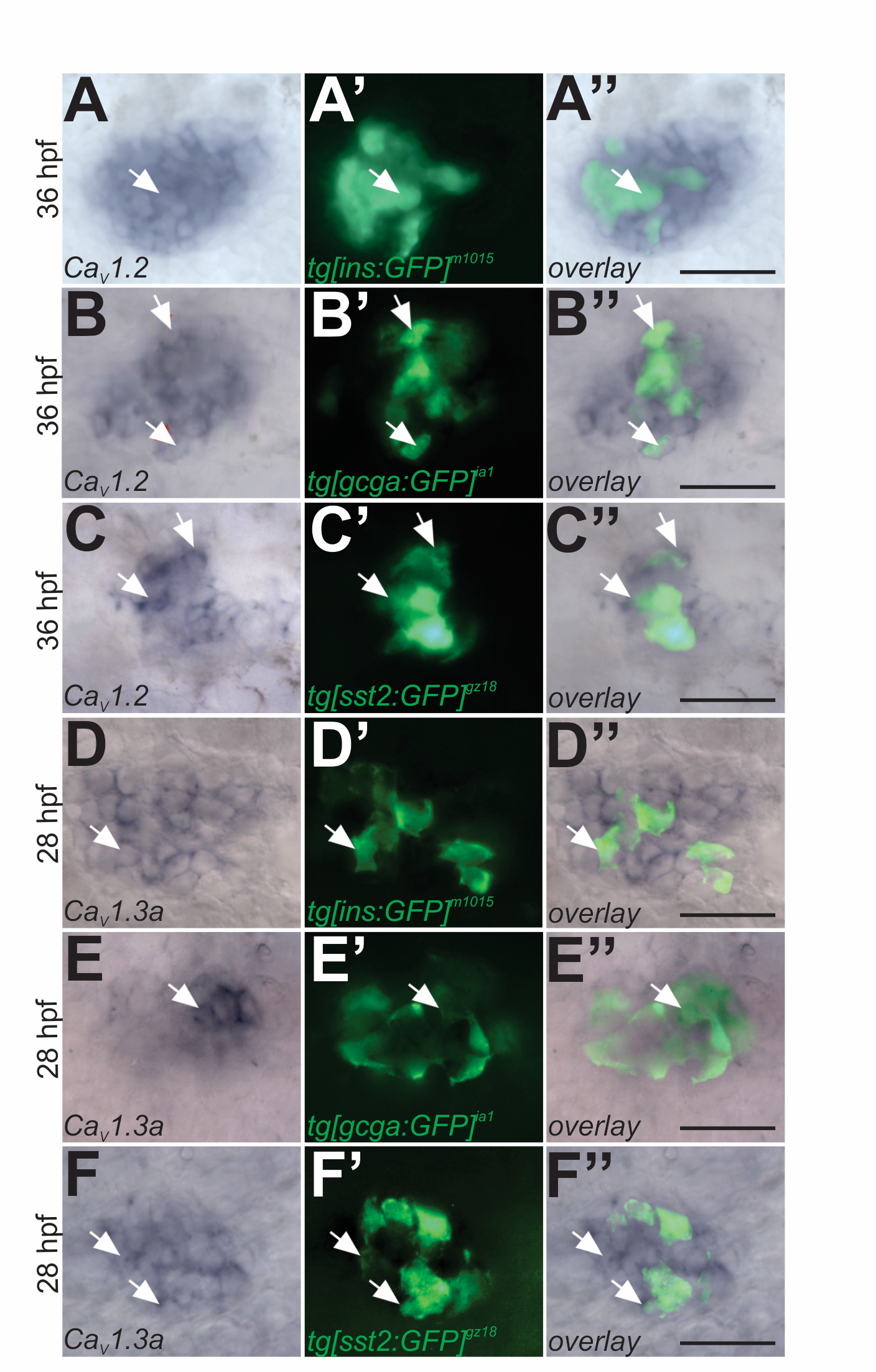


**Supplementary Figure 4:** ***Ca_V_1.2* and *Ca_V_1.3a* are expressed in all endocrine cell types (A, B, C)** Expression of *Ca_V_1.2/cacna1c* mRNA at 36 hpf (hours post fertilization) in relation to GFP in tg[*ins:GFP*]^m1015^ (**A’**) tg[*gcga:GFP*]^ia1^ (**B’**) and tg[sst2:GFP]^gz18^ (**C’**). **(**Overlays are shown in **A’’, B’’, C’’**). **(D, E, F)** Expression of *Ca_V_1.3a/cacna1da* at 28hpf in relation to GFP in tg[*ins:GFP*]^m1015^ (**D’**), tg[*gcga:GFP*]^ia1^ (**E’**) and tg[sst2:GFP]^gz18^ (**F’**). **(**Overlays are shown in **D’’, E’’, F’’**). Arrowheads indicate Ca_V_1.2+ GFP+ or Ca_V_1.3a+ GFP+ cells. Scale bars indicate 30 μm.


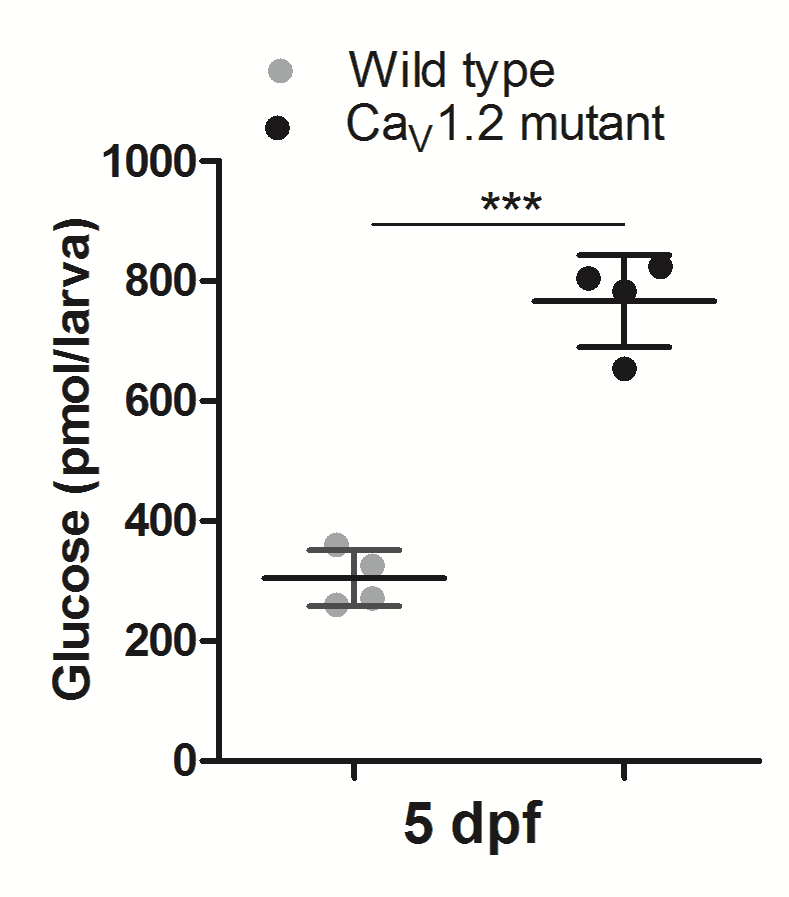


**Supplementary Figure 5:** ***Ca_V_1.2* mutant larvae show elevated glucose level.** Quantification of glucose levels from 5 dpf whole larval extracts (pools of 10 larvae) (n=4 biological replicates) (***p<0.0001, two-tailed t-test). Data are shown as mean values ± s.d.

**Supplementary Figure 6:** ***Ca_V_1.3a* mutant larvae show the same glucose level as the control larvae.** Quantification of glucose levels from 5 dpf whole larval extracts (pools of 10 larvae) (n=3 biological replicates) (*p<0.05, two-tailed t-test). Data are shown as mean values ± s.d.


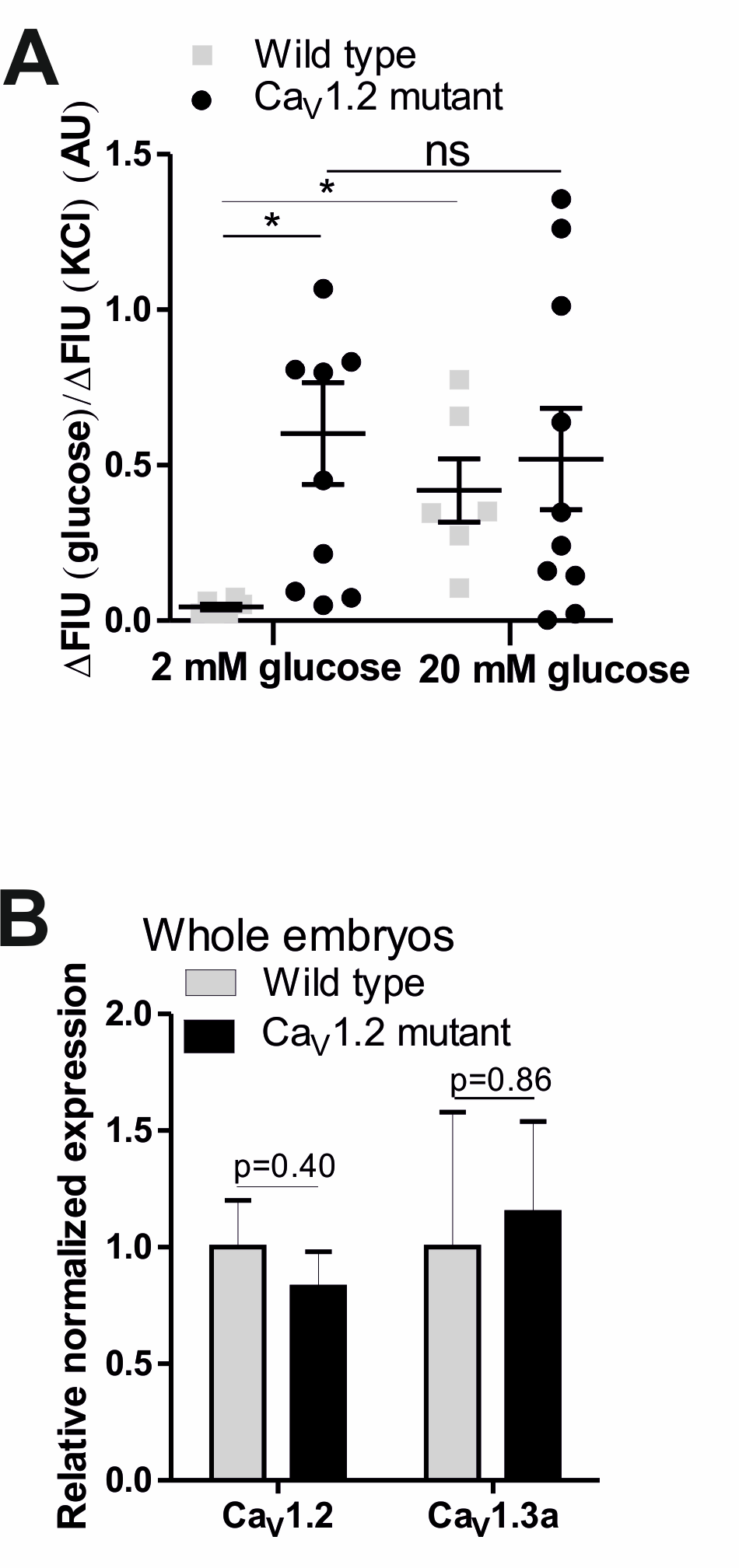


**Supplementary Figure 7: (A)** Glucose-responsiveness of wild type and *Ca_V_1.2* mutant isolated islets, with change in GCaMP6s fluorescence at the indicated concentrations (2 mM or 20 mM glucose) normalized to the total change in fluorescence elicited by KCl depolarization (n=6 and 10 islets, respectively) (*p<0.05, unpaired and paired t-test). Data are shown as mean values ± s.e.m.
