# Supplementary material for: In vivo monitoring of intracellular Ca2+ dynamics in the pancreatic β-cells of zebrafish embryos
Source: Islets. 2018 Dec 6;10(6):221–38. doi: 10.1080/19382014.2018.1540234 (PMC6300091; doi:10.1080/19382014.2018.1540234)
Supplement: Supplemental Material [file kisl-10-06-1540234-s001.zip › Supplementary_Video_description_Lorinczetal.docx]

**Video 1: Intraperitoneal injection of zebrafish larva.** To confirm the intraperitoneal injection of the zebrafish larva, glucose was co-injected with Rhodamine B isothiocyanate-Dextran, and was re-injected without moving the needle, after Ca^2+^ imaging was taken. Time lapse was taken using Leica DM6000B microscope, 5X dry objective. Scale bar indicates 10 μm.

**Video 2**: **D-Glucose response of pancreatic islet in living zebrafish larva.** Time lapse of the islet *in vivo* showing β-cell specific, immediate GCaMP6 signal alteration after intravenous injection of D-glucose (˜10-20 mM final concentration) in 5 dpf larva. Time lapse was taken using Leica DM6000B microscope, 40X water objective. Scale bar indicates 10 μm.

**Video 3 and 4**: ***In vivo* fluorescence recordings of unstimulated *Ca_V_1.2/isl^m458^* mutant and wild-type larvae.** *Ca_V_1.2/isl^m458^* mutant (Video 3) showed significantly higher basal islet Ca_i_ dynamics than in control animal (Video 4). Scale bars indicate 10 μm.
